# Assessment of Tissue Adequacy by EBUS in Conjunction with PET Scan and Operator’s Experience

**DOI:** 10.3390/clinpract12060099

**Published:** 2022-11-20

**Authors:** Nagla Abdel Karim, Asad Ullah, Steven Pulliam, Ahmed Mostafa, Alejandro Aragaki, Audrey Eubanks, Amit Mahajan, Mahmoud Shehata, Sadia Benzaquen

**Affiliations:** 1 Inova Schar Cancer Institute, University of Virginia, Fairfax, VA 22031, USA; 2 Department of Pathology, Vanderbilt University Medical Center, Nashville, TN 37232, USA; 3 Department of Surgery, University of Tennessee Erlanger School of Medicine, Chattanooga, TN 37403, USA; 4 Division Internal Medicine, University of Cincinnati, Cincinnati, OH 45221, USA; 5 Department of Pathology, Medical College of Georgia, Augusta University, Augusta, GA 30912, USA

**Keywords:** lymph nodes, non-small cell lung carcinoma, lymphadenopathy

## Abstract

Mediastinal lymph node assessment is a crucial step in non-small cell lung cancer staging. Positron emission tomography (PET) has been the gold standard for the assessment of mediastinal lymphadenopathy, though it has limited specificity. Endobronchial ultrasound-guided transbronchial needle aspiration (EBUS-TBNA) is quick, accurate, and a less invasive method for obtaining a diagnostic sample in contrast to mediastinoscopy. We performed a retrospective chart analysis of 171 patients to assess the adequacy of tissue obtained by EBUS for diagnosis and molecular profiling as well as the assessment of staging and lymph node (LN) stations diagnostic yield, in correlation to PET scan and the operator’s level of experience. A significantly increased tissue adequacy was observed based on the operators’ experience, with the highest adequacy noted in trained Interventional Pulmonologist (IP) (100%), followed by >5 years of experience (93.33%), and 88.89% adequacy with <5 years of experience (*p* = 0.0019). PET-CT scan ^18^F-fluorodeoxyglucose (FDG) uptake in levels 1, 2, and 3 LN had a tissue adequacy of 76.67%, 54.64%, and 35.56%, respectively (*p* = 0.0009). EBUS bronchoscopy method could be used to achieve an accurate diagnosis, with IP-trained operators yielding the best results. There is no correlation with PET scan positivity, indicating that both PET and EBUS are complementary methods needed for staging.

## 1. Introduction

Mediastinal lymph node (LN) assessment is crucial in the staging and management of non-small cell lung cancer (NSCLC). The extent of involvement of hilar and mediastinal LN plays a major role in the multidisciplinary treatment decisions that range from neoadjuvant therapy, surgical resection, chemoradiation, or eligibility for clinical trials [1,2,3,4].

Pathological staging of the mediastinum through either mediastinoscopy or endobronchial ultrasound bronchoscopy (EBUS) has excellent specificity and sensitivity. Unfortunately, their potential complications, need for anesthesia, and costs make imaging techniques a widely-used initial modality for mediastinal staging for lung cancer patients. A clinical staging positron-emission tomography-computed tomography (PET-CT) scan is the most commonly used imaging modality. PET-CT relies on a standardized uptake value (SUV) calculation compared to normal tissue, thus providing the relative metabolic activity value in an LN. PET-CT is relatively sensitive in identifying metabolically active LNs but has low specificity with pathological diagnoses other than malignancy, such as inflammation and infection [1,5,6,7,8,9,10]. Therefore, CT and PET-CT scans cannot be used alone for staging in lung cancer patients due to the high proportion of false-positive and false-negative rates. Instead, PET-CT imaging needs to be followed by lymph node sampling techniques [11], which is considered the standard of care for the assessment of mediastinal lymphadenopathy patients with cancer [7,8,9].

The American College of Chest Physicians recommends that lung cancer patients treated by surgical resection on a curative basis should be monitored by CT every six months for two years, then annually thereafter [12]. However, radiological modalities are limited when used for lung cancer recurrence confirmation and differentiating between benign and malignant lesions. Lesions mimicking recurrence, such as inflammation, infection, and fibrosis (which may be sequelae of treatment), may appear in CT as parenchymal abnormalities and lymphadenopathy. Additionally, CT and PET-CT exhibited a high false-positive rate for recurrence diagnosis in lung cancer (13–46%) [13,14,15,16]. Consequently, pathological sampling became mandatory for the diagnosis of recurrence and before initiating treatment [17].

The standard of care for mediastinal staging in patients with lung cancer is endobronchial ultrasound-guided transbronchial needle aspiration (EBUS-TBNA). This procedure is minimally invasive, highly effective, and can be used to diagnose lung cancer, infections, and other diseases causing enlarged lymph nodes in the chest. EBUS-TBNA allows for real-time imaging of the airways, lungs, lymph nodes, blood vessels, and the potential to biopsy difficult-to-reach areas through needle aspiration. Compared to mediastinoscopy, EBUS-TBNA is less invasive, can access more lymph node stations for sampling, and has a lower complication profile.

In this study, we reviewed the adequacy of tissue obtained for diagnosis, successful molecular profiling, adequate staging, and diagnostic yield by the EBUS-TBNA at a major academic institution compared to PET-CT imaging. In addition, we analyzed the effect of the operator’s level of experience on the adequacy of the resulting tissue.

## 2. Materials and Methods

This retrospective chart review study included [X] LN stations of 171 patients who underwent EBUS at the University of Cincinnati Medical Center over a period of three years. The study included patients with the diagnosis of advanced stage III and IV NSCLC. Charts were reviewed for pathological diagnosis, EGFR status, LN stations, PET scan results, and EBUS operator experience. The availability of tissue was assessed and pathological diagnosis has been described.

Diagnostic accuracy was measured by the diagnostic yield and the percentage of malignancies found in patients with known metastatic disease.

### 2.1. Inclusion/Exclusion Criteria

Patients must have met all of the following criteria to be included in this study:

1. Diagnosis of advanced NSCLC, stages III or IV;
2. Undergone EBUS at the University of Cincinnati Medical Center;
3. Presence of EGFR status.

### 2.2. Pathalogical Diagnosis and Evaluation of Diagnostic Accuracy

The inclusion criteria of a diagnosis of advanced NSCLC of stage III or IV was considered the diagnostic gold standard. If the tissue sampled were diagnostic, whether positive or negative, for malignant cells, then the EBUS was considered adequate. If the pathology results were inadequate for a diagnosis, then the EBUS was deemed inadequate. Adequacy was determined on a patient level.

### 2.3. Statistical Data Analysis Methods

Success of obtaining a positive biopsy was considered as the endpoint. Chi-square test was used to compare the different levels of operator’s diagnostic accuracy as well as tissue adequacy with FDG uptake levels. Data were analyzed using SAS software Version 9.4 (SAS Ins, Cary, NC, USA).

## 3. Results

### 3.1. Adequacy of Samples Collected

171 patients underwent EBUS and were included in this retrospective study.

Most of the 171 patients who underwent EBUS in our study achieved adequate tissue levels upon sampling for diagnostic purposes (*p* < 0.0001). Of all lymph node (LN) stations tested, LN stations 4R and 4L showed more positive tissue results compared to the other stations, but with no statistical significance. No correlation was found between PET-CT positivity and tissue adequacy for diagnosis by EBUS (*p* = 0.6410).

### 3.2. Effect of Operator Experience

**Figure 1:**
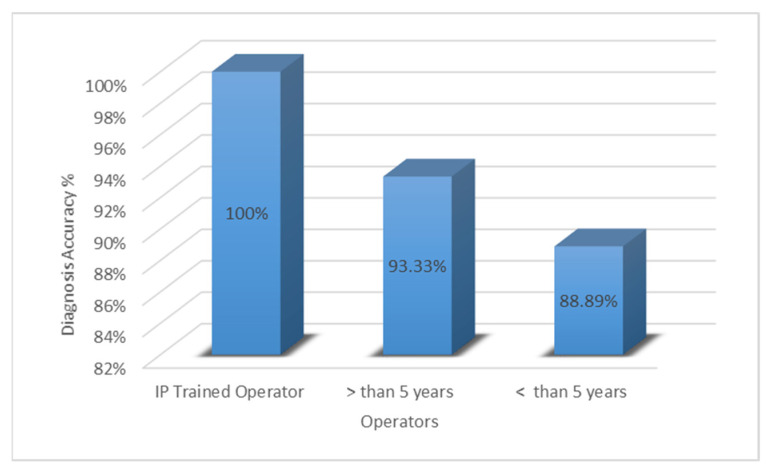
Inter-operator diagnostics accuracy. The diagnostic accuracy (%) is shown on the *y*-axis and the operator experience is shown on the *x*-axis.

Operators who were fellowship-trained in Interventional Pulmonology (IP) achieved the most significant tissue diagnostic accuracy of 100% (*p* = 0.0019). Operators with greater than five years of general experience achieved a diagnostic accuracy of 93.33%. Operators with less than five years of experience obtained an 88.89% diagnostic accuracy (*p* = 0.0019) (Figure 1).

### 3.3. Diagnostic Accuracy Compared to PET-CT

**Figure 2:**
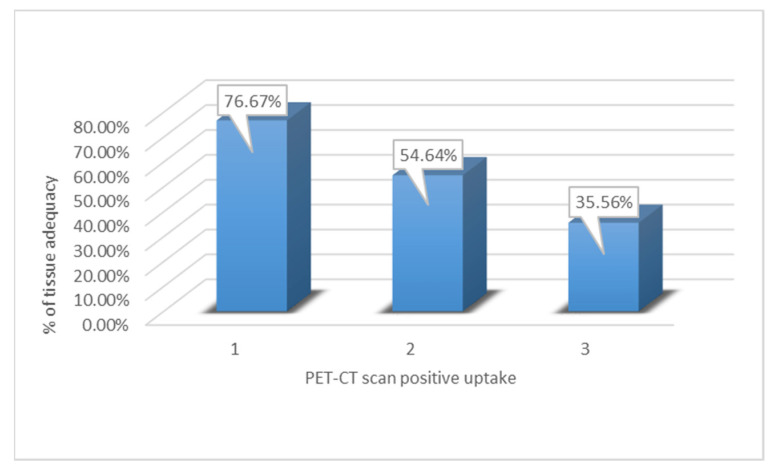
Tissue Adequacy by PET-CT scan: the percentage of tissue adequacy is shown on the *y*-axis and the PET-CT scan positive FDG uptake levels of 1, 2, and 3 are shown on the *x*-axis.

The diagnostic tissue adequacy had a positive correlation with the PET scan when analyzed by an operator with more than five years of experience. The level of ^18^F-fluorodeoxyglucose (FDG) uptake was binned into two classes: 1, 2, and 3. The percentage of tissue adequacy in relation to the PET-CT scan positive uptake depended on the degree of uptake noted in each scan, with ^18^F-fluorodeoxyglucose (FDG) uptake levels of 1, 2, and 3 achieving a tissue adequacy percentage of 76.67%, 54.64%, and 35.56%, respectively (*p* = 0.0009) (Figure 2).

## 4. Discussion

Effective treatment of lung cancer is challenging due to its incidence, high mortality, and inability to diagnose at early stages. While there have been many advances in the surgical treatment of early stage lung cancer, medical treatments are only beginning to see the effectiveness of targeted therapies.

The recent advances in diagnostic techniques have eliminated the need for invasive procedures to diagnose and stage lung cancer. This leap in efficiency has been facilitated by the expanding field of biomarkers and molecular markers that make personalized approaches to cancer treatment possible [18,19,20]. However, these techniques have been challenged by increasing demand for more specificity, sensitivity, and larger pathological samples. In addition, the increasingly large set of available treatment options requires further categorization of diseases to benefit from specific agents and treatment combinations, instead of using treatments against broad disease categories [10,21]. This phenomenon was highlighted by a recent 2021 study that analyzed the efficacy of FDG-PET/CT for detecting malignancies and found a positive predictive value of 98.9%.

Despite this positive result, the authors recommended including biopsies in patient management to properly diagnose genetic mutations and thus avoid inappropriate treatment and misdiagnoses [22]. The 18F-FDG PET/CT method combines metabolic and morphologic information with a relatively high sensitivity and specificity for distinguishing malignancies (96.8% and 77.8%, respectively) [23,24,25]. Consequently, PET/CT is widely used as a sole method for determining staging and appropriateness for surgical resection. This is no longer the standard of care, especially in cases with tumors greater than 3 cm in size, where EBUS-TBNA staging is not preferred [26].

EBUS-TBNA was first described in 1992 and has quickly become the preferred mediastinal lymph node sampling method. The technique proved to be more efficient and accurate, less invasive, and more cost-effective than mediastinoscopy [3]. Its sensitivity and specificity remained superior even with the smaller amount of specimen acquired during the procedure. Based on this, it is the diagnostic modality of choice for pathological staging in patients with lung cancer and mediastinal or hilar lymphadenopathy [11].

An important utility of EBUS-TBNA lies in its ability to obtain adequate tissue quantities to provide a molecular diagnosis in cases of NSCLC. This role cannot be emphasized enough, as specific chemotherapeutics and targeted immunotherapies have been shown to improve progression-free survival [27]. Labarca and colleagues recently performed a meta-analysis examining the adequacy of tissue sampling via EBUS-TBNA for molecular prognostication in cases of commonly reported NSCLC mutations, such as EGFR, ALK, and PD-L1. They reported a sufficient amount of tissue was obtained to analyze for EGFR in 93% of cases and ALK in 92% of cases, with adequate sampling to assess for co-mutations in 94% of cases [28]. Other mutations that have a significant impact and have targeted therapies available, such as PD-L1, have been assessed by Sakakiriba et al. They achieved a 100% tissue adequacy rate in a cohort of 97 samples [29].

The proficiency of the endoscopist is an essential factor due to the complex methodology of EBUS-TBNA and the importance of collecting an adequate diagnostic sample. The American Thoracic Society (ATS), European Respiratory Society (ERS), and the American College of Chest Physicians (CHEST) recommend that an endoscopist should perform at least 40–50 procedures under supervision to acquire initial competence, then 20–25 procedures per year to maintain these skills [30,31], which has been supported in other studies [32]. A 2015 study reported a median of 212 procedures should be performed to obtain the necessary experience and technical skills in EBUS-TBNA [33], which is consistent with the British Thoracic Society recommendations that emphasize the differences in the learning curves of trainees [34]. EBUS-TBNA accuracy in diagnosis significantly relies on the efficiency of the technique used to carry out the procedure. As has been described, training with EBUS-TBNA can be heterogenous, depending on factors like sedation type and lymph node size [35]. Our results are in concordance with these findings showing that the performance of the endoscopist has a pivotal role in the procedure’s results [36,37,38,39].

EBUS for diagnosis of LN stations has been repeatedly investigated in comparison to mediastinoscopy with satisfying sensitivity (81% vs. 79%), specificity (100% vs. 100%), negative predictive value (91% vs. 90%), and diagnostic accuracy (93% vs. 93%) [40]. EBUS-TBNA sensitivity (76.9%, 80.0%, and 92.3%, respectively) and specificity (55.3%, 70.1%, and 100%, respectively) are significantly higher when compared to CT and PET scans [41]. Some studies have used EBUS-TBNA as a reference in evaluating the efficiency of diagnostic imaging techniques such as PET-CT scans [42].

The ability to combine EBUS with rapid on-site evaluation (ROSE) has led to a decreased length of procedures and fewer passes for biopsies. Harangus et al. investigated ROSE in conjunction with EBUS to evaluate the optimization of the procedure. The authors achieved adequate tissue sampling in 100% of their cases when evaluating for EGFR and ALK mutations. They found that ROSE allowed for a highly sensitive (92%) adjunct to EBUS with an equally high positive predictive value (93%) that clinicians could use to predict the final tissue diagnosis in cases of NSCLC [43]. Thus, the utility of ROSE should be further investigated in conjunction with EBUS, as it can increase procedural value by reducing examination length, reducing the number of associated biopsies needed, permitting rapid preliminary diagnosis, and securing adequate tissue samples.

One aspect of EBUS that requires further study is the standardization, or lack thereof, associated with the procedure. There are currently no defined criteria by which tissue adequacy can be repeatedly determined. A retrieved tissue sample is generally considered “adequate” when a diagnosis can be made, but this relative term leaves room for interpretation and lack of uniformity. Thus, efforts should be made to standardize the definition of “adequate” regarding tissue sampling to allow for a more congruent implementation across the clinical and scientific community.

### Limitations

The main limitation of our retrospective study was the inadequate data on molecular profiling that has been the cornerstone for treatment decisions in patients with advanced NSCLC. We plan further studies to assess tissue adequacy in relation to the full panel of molecular profiling in a subset of patients.

## 5. Conclusions

Most patients who underwent EBUS had adequate tissue samples available for diagnosis. Since no correlation was found between PET positivity and tissue adequacy for diagnosis by EBUS, we recommend that PET scans and EBUS-TBNA should be used in a complementary manner for proper diagnosis and staging in patients with NSCLC. Regarding tissue adequacy, the most reliable samples were attained by an IP-trained operator, followed by an operator with more than five years of experience. Our results underscore the importance of employing a highly trained operator, particularly one with a specific focus on the biopsied organ.

As discussed previously, the efficiency of EBUS in achieving adequate tissue for pathological and molecular diagnosis has improved over the years and makes it an incredibly valuable procedure. Given the shift in the management of lung cancer to a genetically focused, personalized approach, greater emphasis should be put on EBUS as a tool to diagnose lung cancer patients more accurately and to subsequently administer therapies targeted to the genetic abnormalities found in their particular cancer.

## Data Availability

Data is available on request from corresponding author.
